# Genome Scan for Locus Involved in Mandibular Prognathism in Pedigrees from China

**DOI:** 10.1371/journal.pone.0012678

**Published:** 2010-09-10

**Authors:** Qin Li, Feng Zhang, Xin Li, Fengshan Chen

**Affiliations:** 1 Department of Orthodontics, Dental School, Tongji University, Shanghai, China; 2 Beijing Institute of Genomics, Chinese Academy of Sciences, Beijing, China; National Institutes of Health, United States of America

## Abstract

**Background:**

It is well known that genetic components play an important role in the etiology of mandibular prognathism, but few susceptibility loci have been mapped.

**Methodology:**

In order to identify linkage regions for mandibular prognathism, we analyzed two Chinese pedigrees with 6,090 genome-wide single-nucleotide polymorphism (SNP) markers from Illumina Linkage-12 DNA Analysis Kit (average spacing 0.58 cM). Multipoint parametric and non-parametric (model-free) linkage analyses were used for the pedigrees.

**Principal Finding:**

The most statistically significant linkage results were with markers on chromosome 4 (LOD  = 3.166 and NPL = 3.65 with rs 875864, 4p16.1, 8.38 cM). Candidate genes within the 4p16.1 include EVC, EVC2.

**Conclusion:**

We detected a novel suggestive linkage locus for mandibular prognathism in two Chinese pedigrees, and this linkage region provides target for susceptibility gene identification, a process that will provide important insights into the molecular and cellular basis of mandibular prognathism.

## Introduction

Mandibular prognathism (MP) is a common clinical problem all over the world. However, its prevalence varies relative to populations: the highest incidence has been observed in Asian populations (approximately 15%) and the lowest in Caucasian populations (1%)[1,2]. The uinque concave profile of mandibular prognathism not only seriously affects the masticatory function but also extremely endangers psychology to patients. Today, this type of disharmony remains difficult for dentists because of varied etiologies and limited understanding of the mandibular growth[3].

It is well known that environmental and genetic components have both contributed to the etiology of mandibular prognathism[4]. Various environmental etiologies, eg. imbalances in the endocrine system and hormones[5], enlarged tonsils[6] have been reported to be involved in the forming of mandibular prognathism. However, there is great interest in the genetic component of the etiology and numerous studies suggest that genetic components play an important role in its etiology[7]–[10]. But the inheritance pattern of mandibular prognathism is heterogeneous, findings have been reported suggesting autosomal-recessive inheritance, autosomal-dominant inheritance, dominant inheritance with incomplete penetrance or a polygenic model of transmission[11]. Recently Cruz RM[1] examined data on 55 extended families with at least one affected member with MP and performed a complex segregation analysis to access the inheritance pattern. It turned out that the majority of the pedigrees suggested autosomal dominant inheritance.

Recent progress in molecular genetics has enabled the genetic determinant to be approached directly. Genetic linkage maps using various types of polymorphic markers are essential tools in many genetic studies. Short tandem repeat (STR) become popular genetic markers because of their polymorphism and hereditary. Yamaguchi et al[12] and Frazier-Bowers et al [13] performed genome-wide linkage analysis with STR respectively and found out some mandibular prognathism susceptibility loci ----1p36, 6q25, 19p13.2 [12] and 1p22.1, 3q26.2, 11q22, 12q13.13 and 12q23 [13].

As ethnicity is a major risk factor for MP and Chinese pedigrees have never been studied, we speculated as to the existance of a special locus for Chinese Han People. In addition, technological advances in genotyping Single Nucleotide Polymorphisms (SNPs) has caused an increase in their application in linkage mapping studies because of high-throughput. In this study, we recruited two MP pedigrees of Chinese Han People in an attempt to discover a specific locus or the major gene that regulates mandibular growth by performing a genome-wide analysis with SNPs.

## Results

### Phenotypic characterization (cephalometric analysis)

When compared with normative cephalometric standards of China[14], no subjects had undergrowth of maxilla relative to normal maxillary length (ANS-Ptm, anterior nasal spine-pterygomaxillary fissure). Three subjects were under 18 years old and were diagnosed as affected individuals with a negative ANB angle and normal maxillary length. The cephalometric variables that differ between affected and unaffected individuals are shown in Table 1.

**Table 1:** The cephalometric variables that differ between affected and unaffected individuals.

cephalometric measure	Affected members		Unaffected members		Norms	
	Mean	SD	Mean	SD	Mean	SD
SNA	81.34	3.03	84.32	4.16	82.8	4.1
SNB	84.47	2.93	80.37	2.34	80.1	3.9
ANB	−3.13	2.64	4.05	2.13	2.7	2.1
Wits(mm)	−6.25	3.72	3.14	2.67	−1.2	2.5
ANS-Ptm(mm)	52.49	2.86	54.36	3.24	51.1	2.6
Co-Po(mm)	117.18	3.03	107.47	3.79	110.2	3.8

Footnotes:

*, cephalometric standards of China.

ANB, anteroposterior relationship of the maxilla and mandible.

SNA, anteroposterior maxillary position to anterior cranial plane.

SNB, anteroposterior mandibular position to anterior cranial plane.

Wits (mm), length of AO-BO distance.

ANS-Ptm (mm), maxillary unit length.

Co-Po (mm), mandibular unit length.

### Genome-wide linkage analysis

**Figure 1:**
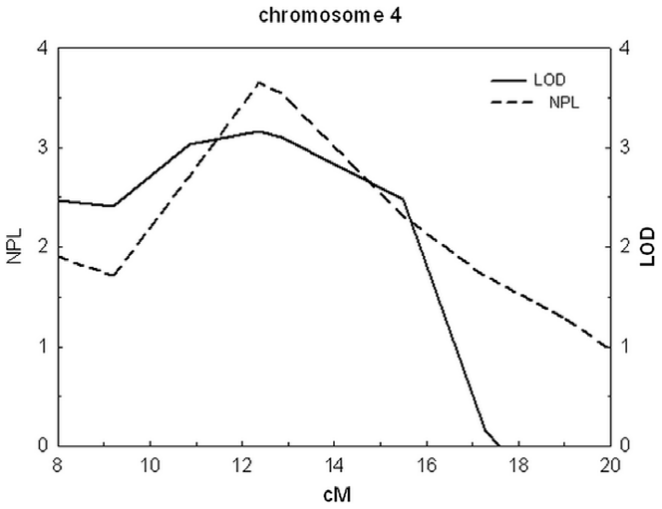
Chart of LOD and NPL value on 4p16.1. X axis indicates position from p terminus of chromosome (cM). Dashed line indicates LOD scores, and solid line indicates NPL scores. LOD: logarithm (base 10) of odds. NPL: value of non-parametric linkage analysis.

Pedigree analysis by visual inspection suggested an autosomal-dominant inheritance with incomplete penetrance. Multipoint parametric and non-parametric linkage scores obtained from the 0.58-cM resolution genome-wide scan revealed that only one chromosomal locus provided evidence of linkage: 4p16.1 (LOD = 3.166 and NPL = 3.65 with rs 875864, 4p16.1, 8.38 cM). The multi-point linkage results on chromosome 4p16.1 are shown in Figure 1 and Table 2. Both of LOD values of the respective pedigree were larger than 1, which means both of the pedigrees showed linkage to this region and no genetic heterogeneity was observed between the two pedigrees.

**Table 2:** The multi-point linkage results on4p16.1.

locus	marker	position from pter(cM)	NPL	LOD
4p16.1	rs934498	7.11	2.01	2.496
4p16.1	rs920683	7.16	2.03	2.503
4p16.1	rs873924	7.18	2.03	2.503
4p16.1	rs2654498	9.21	1.71	2.403
4p16.1	rs726111	10.85	2.71	3.308
4p16.1	rs875864	12.37	3.65	3.166
4p16.1	rs7658616	12.46	3.63	3.156
4p16.1	rs875579	12.86	3.54	3.106
4p16.1	rs1173466	15.49	2.31	2.745

Footnotes:

Locus: map position as identified by the marker.

cM: centiMorgans.

pter: p terminus of chromosome.

LOD: logarithm (base 10) of odds.

NPL: value of non-parametric linkage analysis.

These data substantiated the linkage signals of the susceptibility locus on 4p16.1 from our MP pedigrees. We futher identified candidate genes of biologic interest for the locus using biologic approaches (http://www.ncbi.nlm.nih.gov). This search revealed that human genes EVC, EVC2 are within this region.

## Discussion

Identification of genetic susceptibilities to MP is the first step toward understanding the molecular pathogenesis of MP. Although the previous studies [12], [13] have determined some loci susceptible for MP, we found out a novel locus in Chinese Han People with slightly higher NPL and LOD scores.

There are various skeletal types of Class III malocclusion, such as mandibular protusion, maxillary retursion or a combination of both[15]. To minimize the possibility of heterogeneity, we chose one ethnicity and focused on one subtype of skeletal Class III— mandibular prognathism. In this study, the genetic marker—SNPs we used were different from STR which were used in previous similar studies[12], [13]. Eventhough the genetic polymorphism of SNPs cannot be compared to STR, an increased number of markers (6090), which are evenly spaced and cover a high proportion of the genome, can make up for this low-variability deficiency. In addition, compared to STR, SNP genotyping platforms are almost fully automated and error rates tent to be much lower which improves accuracy of genetic map[16]. As the markers we used in this study were dense enough, with an average 0.58 cM genetic map spacing and an average 441 kb physical map spacing, a fine-mapping with more markers in the region of interest was unnecessary.

In searching for an genetic linked region, it is important to distinguish between pointwise (also called nominal) significance levels and genome-wide significance levels. As multipoint parametric and non-parametric (model-free) multipoint linkage analyses were performed, we identified susceptable locus according to both these results— the value of NPL and LOD. The linkage analyses results revealed a new locus— 4p16.1 (LOD = 3.166 and NPL = 3.65 with rs 875864, 4p16.1, 8.35 cM). Taking into account for both values, we considered 4p16.1 as a novel locus genetic linked to MP.

There are 23 function genes in the region. It harbors positional candidate genes of interest such as EVC and EVC2. EVC2 encodes a protein that functions in bone formation and skeletal development. Mutations in this gene[17], [18], as well as in a neighboring gene—EVC which lies in a head-to-head configuration, cause an autosomal recessive skeletal dysplasia that is also known as chondroectodermal dysplasiab or cause acrofacial dysostosis Weyers type, a disease that combines limb and facial abnormalities. As the mutations in these genes always cause skeletal dysplasia, we suggest that some novel mutations in EVC2 and EVC may be relevant to the form of mandibular prognathism.

In summary, when performing a genome-wide linkage analysis with two mandibular prognathism pedigrees of Chinese Han people, we detected a novel chromosomal region—4p16.1 potentially linked to mandibular prognathism. EVC2 and EVC were considered as candidate genes to influence the bone tissue. To efficiently identify the mutations that may be responsible for this disease, second-generation sequencing with Solelxa technology will soon be carried out within this candidate region. The findings in the current study can be combined with those from previous studies to further understand the genetic basis of mandibular prognathism.

## Materials and Methods

### Disease Criteria and Pedigree Recruitment

The protocols for the current study, as well as participant consent, were reviewed and approved by the institutional review board at Tongji University Dental School. Consent to participate in this study (including a release for dental records) was obtained from every adult participant or in the case of minors from a parental guardian. The consent was written.

**Figure 2:**
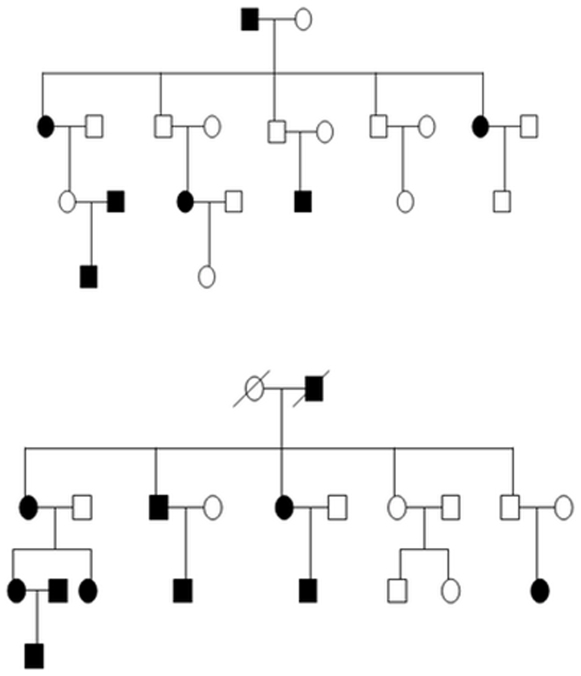
The pedigree chart of mandibular prognathism families. The mandibular prognathism samples are indicated by darkened (affected) circles or squares. The squares indicate male; the circles indicate female; the diagonal lines indicate deceased family members.

The family structure was as follows: pedigrees comprised of 4 generations with a total of 42 individuals containing 18 affected individuals. The age range was between 12 and 64 years, the average age being 38.7 years. All of the samples were drawn from China. These two pedigrees were not biologically related and were from different provinces. The pedigree charts were shown in Figure 2.

The subjects were first diagnosed by lateral cephologram, in conjunction with orthodontic study models or visual inspection. Subjects were diagnosed as affected individuals if they had an ANB angle (Point A-Nasion-Point B) of centric jaw relationship under 0.0 degrees[1], [12] and a negative Wits appraisal greater than −2.0 mm [3]. The total length of the mandible was evaluated by the Condylion-Pogonion distance. No subjects had severe undergrowth of maxilla relative to normal maxillary length (ANS-Ptm, anterior nasal spine-pterygomaxillary fissure). Three subjects were under 18 years old and were diagnosed as affected individuals with a negative ANB angle and normal maxillary length. The dentofacial phenotype of the deceased individuals was confirmed by their offsprings. None of the participants had a congenital disorder such as cleft palate or physical diseases.

### Cephalometric Analysis

**Figure 3:**
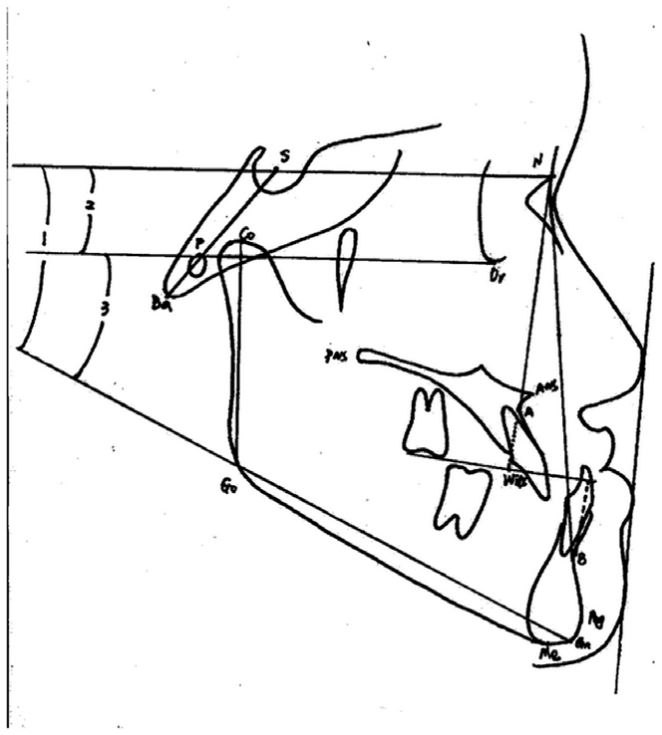
Cephalometric tracing and major measurements. Co-Gn (mm), mandibular unit lengh; Go-Gn (mm), mandibular corpus lengh; SN (mm) anteroposterior lengh of cranial base; wits (mm), length of distance AO-BO; ANB, anteroposterior relation of maxilla and mandible; SNA, anteroposterior maxillary position to anterior cranial plane; SNB, anteroposterior mandibular postion to anterior cranial plane; SN to GoGn (angle1), inclination of SN to mandibular plane GoGn; SN to FH (angle 2), inclination of SN to FH plane; FH to GoGn (angle 3), inclination of FH to mandibular plane GoGn.

The cephalometric tracing were performed by at least two orthodontists. Eighteen cephalometric parameters were used for diagnosis of the samples. Cephalometric tracing and major measurements were followed as figure 3.

### Power Estimation

The power to detect linkage was calculated using the program SLINK with 500 replicates. This pedigree showed an maximum LOD score of 4.5, assuming an autosomal-dominant model with incomplete penetrance of 0.95[19] and a phenocopy rate of 0.005. The power to detect LODs scores greater than 2 and 3 were 75% and 41%, respectively.

### Genotyping and Linkage Analysis

Genomic DNA was isolated from whole blood cells with the use of a QIAamp DNA Blood Kit (QIAGEN GmbH, Hilden, Germany). Genotyping was carried out with Illumina Linkage-12 DNA Analysis Kit (Human) (Illumina, San Diego, USA). A Genome scan was performed with a total of 6090 single-nucleotide polymorphism (SNP) markers, having average 0.58 cM genetic map spacing and average 441 kb physical map spacing. The average minor allele frequency observed in the Asian sample group was 0.28.

Multipoint parametric and non-parametric (model-free) multipoint linkage analyses were performed on the family of the subjects. Non-parametric linkage (NPL) analysis, which has been described as a powerful approach compared with the commonly used parametric methods[20], was run to account for the possibility of alternative modes of inheritance. As the literature strongly favors an autosomal-dominant inheritance of mandibular prognathism, and our pedigree analyses by inspection also suggested this pattern, we assumed an autosomal-dominant inheritance with incomplete penetrance of 0.95 and phenocopy rate of 0.005 for the parametric analysis and an affected allele frequency of 0.0001 via the program MERLIN version 1.01[21]. The reported linkage scores were from the joint analysis of the two families. Marker allele frequencies were estimated from the founders of the pedigree via MERLIN. Mendelian inconsistencies of the genotype data were investigated with Pedcheck version 1[22] and improper genotypes were set to missing before the linkage analysis.
